# Psilocybin enhances insightfulness in meditation: a perspective on the global topology of brain imaging during meditation

**DOI:** 10.1038/s41598-024-55726-x

**Published:** 2024-03-26

**Authors:** Berit Singer, Daniel Meling, Matthias Hirsch-Hoffmann, Lars Michels, Michael Kometer, Lukasz Smigielski, Dario Dornbierer, Erich Seifritz, Franz X. Vollenweider, Milan Scheidegger

**Affiliations:** 1 Department of Adult Psychiatry and Psychotherapy, Psychiatric University Clinic Zurich and University of Zurich, Zurich, Switzerland; 2 Department of Psychosomatic Medicine and Psychotherapy, Medical Center - University of Freiburg, Freiburg, Germany; 3 Department of Neuroradiology, University Hospital Zurich, Neuroscience Center Zurich (ZNZ), University of Zurich and ETH Zurich, Zurich, Switzerland

**Keywords:** Computational neuroscience, Psychology, Mathematics and computing

## Abstract

In this study, for the first time, we explored a dataset of functional magnetic resonance images collected during focused attention and open monitoring meditation before and after a five-day psilocybin-assisted meditation retreat using a recently established approach, based on the Mapper algorithm from topological data analysis. After generating subject-specific maps for two groups (psilocybin vs. placebo, 18 subjects/group) of experienced meditators, organizational principles were uncovered using graph topological tools, including the optimal transport (OT) distance, a geometrically rich measure of similarity between brain activity patterns. This revealed characteristics of the topology (i.e. shape) in space (i.e. abstract space of voxels) and time dimension of whole-brain activity patterns during different styles of meditation and psilocybin-induced alterations. Most interestingly, we found that (psilocybin-induced) positive derealization, which fosters insightfulness specifically when accompanied by enhanced open-monitoring meditation, was linked to the OT distance between open-monitoring and resting state. Our findings suggest that enhanced meta-awareness through meditation practice in experienced meditators combined with potential psilocybin-induced positive alterations in perception mediate insightfulness. Together, these findings provide a novel perspective on meditation and psychedelics that may reveal potential novel brain markers for positive synergistic effects between mindfulness practices and psilocybin.

## Introduction

Meditation and psychedelics have attracted increasing scientific interest in recent years^1–4^. While research on the neurophysiology of meditation and psychedelics has grown rapidly, these topics have been studied mainly from the perspective of functional connectivity, resting-state networks, and signal variability, including measures of entropy and criticality^5–7^. This article targets an alternative approach in which a novel topological data analysis (TDA) method is applied^8–11^ to the neurophysiological study of the synergistic effects of meditation and psychedelics.

Meditation can be understood as a form of mental training^12^ with various aims, including improving cognitive and emotional self-regulation^13^, changing attitudes toward the self and others^14^, including their underlying duality^15,16^, and cultivating positive emotional states^17^. Different types of meditation can be distinguished^18,19^. Within the attentional family of meditation, two well-researched attentional practices are focused-attention (FA) practices and open-monitoring (OM) practices. FA involves narrowing of the attentional scope. OM, in contrast, involves releasing attentional control and bringing awareness to moment-to-moment experiential content^18,20^.

Psychedelics are a broad class of consciousness-modulating substances that induce altered states of perception, cognition, emotion, and the sense of self^21–25^. Classical serotonergic psychedelics include lysergic acid diethylamide (LSD), psilocybin, mescaline, and N,N-dimethyltryptamine (DMT).

Meditation has been shown to improve mental health and psychological well-being in many randomized controlled studies^26,27^. Similarly, expanding research on psychedelics has demonstrated their therapeutic potential in treating a variety of psychiatric conditions^3,4,28,29^ such as major depressive disorder^22,30–32^, cancer-related anxiety and existential distress^23,33,34^, and substance use disorders^35,36^. In addition to these potential beneficial effects, an increasing amount of evidence indicates potential adverse effects related to meditation practice^37–40^. The potential adverse effects psychedelics have been emphasized for decades, leading to a recent debate as to whether the persistent negative perceptions of psychological risks from psychedelics use are unsupported by the currently available scientific evidence^41^. In response to recent discussions of a potential hype around both meditation^1^ and psychedelics^42^, researchers in both fields suggest that a balanced view carefully considering both their benefits and their risks is needed.

Interestingly, intriguing evidence of similarities in the phenomenology and neurophysiology of meditation and psychedelics has been found^43–46^. Specifically, recent research has focused on their similarities in altering self-consciousness^43,47^. For instance, high doses of psychedelic drugs and advanced forms of meditation can induce strong, temporary yet qualitatively distinct alterations in one’s sense of self^43^. Moreover, enhanced mood and social skills as well as increased brain plasticity, as indicated by increased levels of brain-derived neurotrophic factor (BDNF), were commonly found in both meditation practitioners and in individuals after the administration of psychedelics^44^. Meditation and psychedelics are both associated with certain neurophysiological changes, including reduced activation in the DMN and increased functional connectivity between DMN structures and certain task-positive networks involving the dorsolateral prefrontal cortex^43,46,48–50^. Notably, there is increasing evidence that psychedelics acutely increase the entropy of spontaneous brain activity (examples include^51–55^, for an overview, see^6,56^), which is hypothesized to index the informational richness of the induced consciousness state^6^. While entropic measures have also been utilized in the literature in the context of meditation, there are discrepacies^57–59^ in the reported findings and future research is needed to allow robust interpretation^60^.

In addition to these similarities, there are notable differences between meditation and psychedelics. Meditation and psychedelics were associated with different effects on the neural core networks and executive functioning^44^. Moreover, psychedelics may prompt substantial therapeutic experiences but are limited in sustaining therapeutic change, whereas meditation requires extensive engagement but provides increasing sustainable benefits over time^45^, suggesting that meditation and psychedelics are complementary.

Recent reviews suggest that meditation and psychedelics exhibit similarities and complementary differences, which may indicate potential synergistic effects when combined^44,45^.

Synergistic effects between psychedelics and meditation are supported by preliminary evidence^44,45,61,62^. In two randomized controlled trials, the use of psilocybin was shown to increase the depth of meditation practice, as indicated by enhanced postintervention mindfulness and increased psychosocial functioning at the 4-month follow-up^7^, as well as greater improvements in psychological well-being and spiritual experiences at a 6-month follow-up^63^.

While these initial studies exemplify the potential synergies between psychedelics and meditative practices, the specific mechanisms of these synergistic effects are not yet sufficiently understood, especially given the broad diversity of meditative and psychedelic states. However, the relationships and interactions between different types of meditation and psychedelic experiences, such as the differential effects of psilocybin on FA vs. OM and attentional stability vs. insightfulness, remain to be further elucidated.

Different neurophysiological structures can be derived from spatiotemporal neural activity, such as functional connectivity, resting-state networks and signal variability. More holistic approaches depicting the overall organization of spatiotemporal brain activity have not yet been applied to explore psychedelic or meditative states. Holistic representations of brain imaging data can be generated using topological data analysis (TDA). Specifically, using mathematical concepts, TDA simultaneously incorporates the global topology and local geometry of high-dimensional data into interpretable representations. Other common advantages of TDA methods include their robustness to noise and independence of the choice of scale, which makes them versatile for data exploration^64,65^. TDA is complementary to conventional methods and has the power to reveal the structure of data, that is latent in other methods^66–68^. For example, TDA of brain imaging data was used to reveal how the modulatory effects of psilocybin in healthy participants were characterized by the appearance of many transient structures of low stability and a small number of persistent structures^69^ in correlation networks. In another study, persistent homology was used to construct a representative state-transition network analyzing the effects of propofol and ketamine on brain dynamics and distinguishing them from individuals with normal consciousness during waking^70^. The latter example remains, to our knowledge, the only application of TDA methods to functional magnetic resonance imaging (fMRI) studies of psychedelic effects.

A particularly promising approach^8^ uses the TDA-based algorithm Mapper^71^ to generate a graphical representation of the dynamic organization of brain states during various cognitive tasks. The resulting *Mapper shape graph* can be thought of as a map of the landscape of brain states at the single-participant level. After generating this graphical representation of overall topology (global and local, spatial and temporal structure) of brain activity, graph theory and topological descriptors can be used to quantify the organizational principles or topography of the Mapper shape graphs. Notably, graph measures were shown to relate to cognitive task performance^8^. In the landscapes of resting states^9^, centrally located (high *degree and closeness centrality*) nodes, were characterized by a uniform ratio of activity in resting-state networks (RSNs). These centrally located nodes represent temporally recurring brain states with equal activity of all canonical RSNs and serve as putative “transition states” between the peripheral nodes, which represent (subject-specific) brain states with one or more dominant RSNs. Across all participants, the variance in mean activation across RSNs was found to continuously decrease toward the center of the graph revealing a “topographic gradient” of the landscape. These findings^8,9^ show that the topological landscapes of resting-state brains reveals novel, relevant information about the organizational principles of brain activity. Another particularly promising tool for studying the toplogical landscape is the *optimal transport (OT) distance*, which allows the study of pairwise similarities, relationships and dependencies between two groups of brain states^11^. There is evidence that OT distance tends to separate tasks with low cognitive load (e.g. video watching or resting state) from tasks with high cognitive load (e.g. working memory and mathematics)^11^. Moreover, OT distance tends to be smaller between tasks that involve similar whole-brain configurations; accordingly, a correlation with task performance is expected^11^.

Therefore, for the first time, we propose the use of topological data analysis to study the overall organization of spatiotemporal brain activity during meditation before and after a five-day psilocybin-assisted meditation retreat in 36 experienced meditators^7,72^ using the Mapper approach^8^. More precisely, an exploratory study of the topological landscape of meditative brain states (i.e. mapping and quantifying the relationships and dependencies of meditative brain states on the level of brain activity) and their potential associations with subjective experience, could provide novel insights into synergistic and potentially therapeutic effects of meditative and psychedelic states. Due to the expected increased entropy of spontaneous brain activity as induced by psychedelics^6^, we hypothesize that psilocybin modulates the organization of the landscape of meditative brain states, and that thereby the Mapper approach will reveal modulatory effects of psilocybin on the relations and dependencies (in terms of topological graph measures) of functional brain activity and psychometric ratings underlying FA, OM and the resting state (RS) and on the overall organization of brain activity.

We also hypothesize that organizational principles of the landscape of meditative brain states can be associated with concepts relating to the subjective experience (e.g., openness, focus, ego dissolution) of different types of meditation. Quantifying organizational principles within this landscape using topological descriptors, such as centrality and OT distances, could reveal novel measures at the level of brain activity that correlate with psychological change processes, such as increased meta-awareness, insightfulness, and positively experienced derealization, all of which may have therapeutic potential in psychedelic-assisted treatment interventions.

## Results

Thirty-six experienced meditators completed two fMRI brain imaging sessions one day before and after a 5-day psilocybin-assisted meditation retreat following a double-blind, randomized, placebo-controlled, parallel-group study design. Placebo or psilocybin (315${\mu \hbox {g}}/\hbox {kg}$ body weight; absolute dose, 21.82±3.7 mg) was administered on the fourth day of the retreat, and the 11D-ASC questionnaire^73^ for ratings of altered states of consciousness (see Methods ) was administered 360 minutes after drug intake as a retrospective measure of subjective effects. Each scan session (one day before and one day after retreat) consisted of 21 min of resting-state, focused attention, and open monitoring sequences (fixed order), with each meditation lasting 7 min. For each subject both scan sessions were concatenated (with transition times excluded) and Mapper shape graphs were generated and analyzed (see Methods and Statistics below).

**Table 1:** Notations used for the Mapper shape graphs and the figures, as well as for the statistics section and the discussion. For precise definitions, refer to the Methods section.

Object	Description
1,2	Sessions of fMRI measurements; 1 = (1 day) preretreat, 2 = (1 day) postretreat
MS = $\{$FA1, OM1, RS1, FA2, OM2, RS2 $\}$	Mediation states = conditions $\times$ sessions (pre/post) $= \{$ FA, OM, RS $\}$ $\times$ $\{1,2\}$
dc(FA*), dc(OM*), dc(RS*)	Degree centrality as functions of subject and session *$\in \{1,2\}$
cc(FA*), cc(OM*), cc(RS*)	Closeness centrality as functions of subject and session *$\in \{1,2\}$ (omitted from the figures, due to a high correlation with DC )
diam(FA*), diam(OM*), diam(RS*)	Diameters as functions of subject and session *$\in \{1,2\}$
d(FA*,OM*), d(FA*,RS*), d(OM*,RS*)	OT distances as functions of subject and session *$\in \{1,2\}$
d(*1,*2)	OT distances as functions of subject and meditation state *$\in \{$ FA, OM, RS $\}$

**Figure 1:**
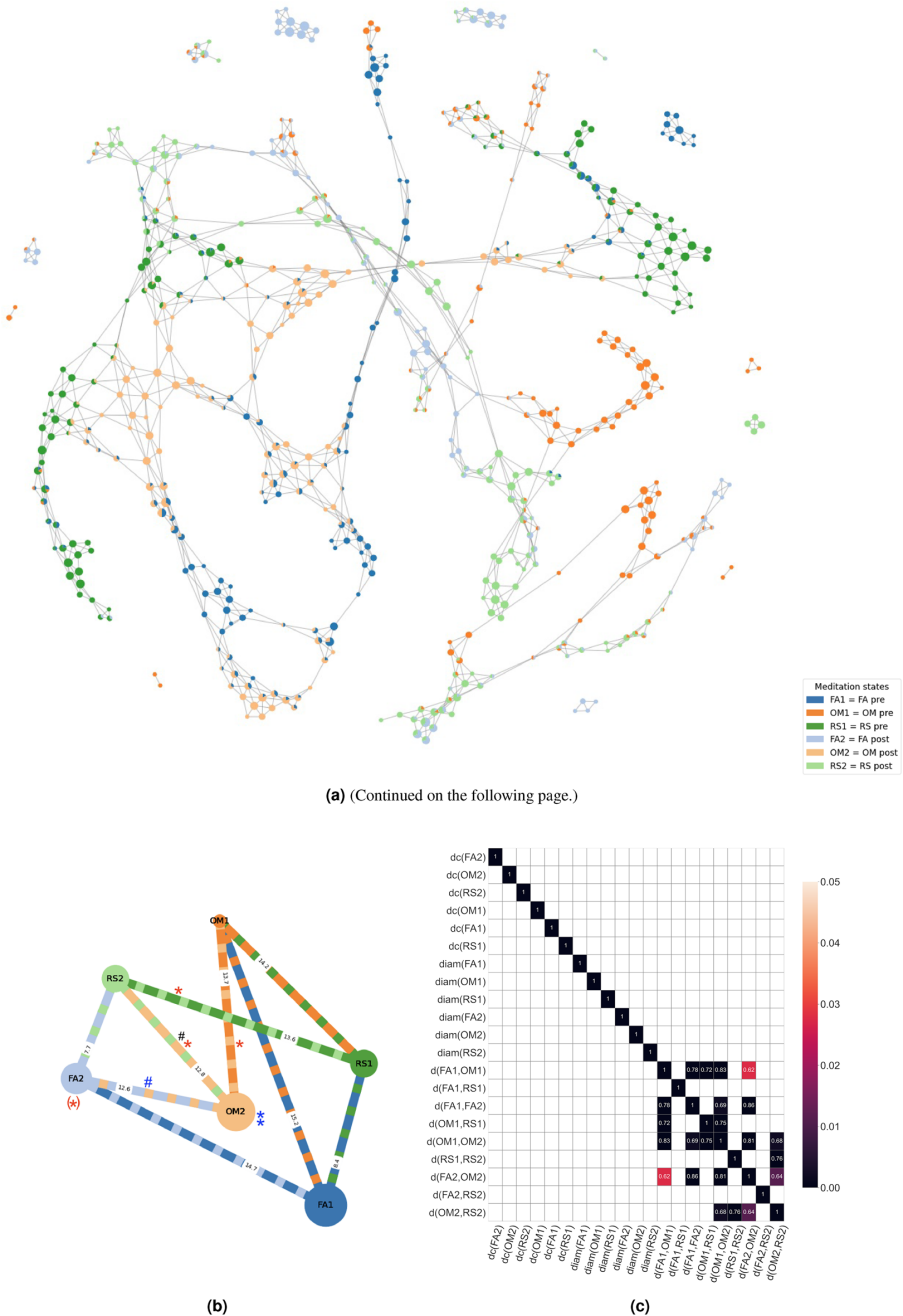
**(a)** A representative Mapper shape graph of one subject (5119) produced with the visualization library DyNeuSR^10^. Each color represents the distribution of an MS (Table 1) in the subject-specific landscape of brain states. **(b)** The network is a schematic picture of the average topological organization of Mapper shape graphs that are shared across all subjects. The nodes are labeled according to the six meditation states (MS), (Table 1). For example, we found that the closeness centrality of the OM significantly increased postretreat, which is represented by the central position of the OM post-node. The length of the edges represents the average OT distance (over all subjects) between the MSs. For example, the OT distance between FA2 and OM2 is smaller than the OT distance between FA1 and OM1. Finally, the diameter of the node represents the diameter of the corresponding MS. Significant increases in the measurements due to retreat and drug effects are annotated by blue and red stars, respectively. Similarly, decreases are annotated by blue and red accidentals, respectively. The star in the bracket near node FA2 indicates that the effect is most likely neglectable. Two stars near OM2 indicate that two measurements (diameter and centrality) were significant. Black accidentals indicate a significant decrease in the distance for the group with low positive derealization. The detailed statistical descriptions are given below. **(c)** Pearson correlations of all graph measures with p values according to the color bar (corrected for 21*21 multiple comparisons). This Figure illustrates how the OT distances are strongly colinear (for topological reasons).

**Figure 2:**
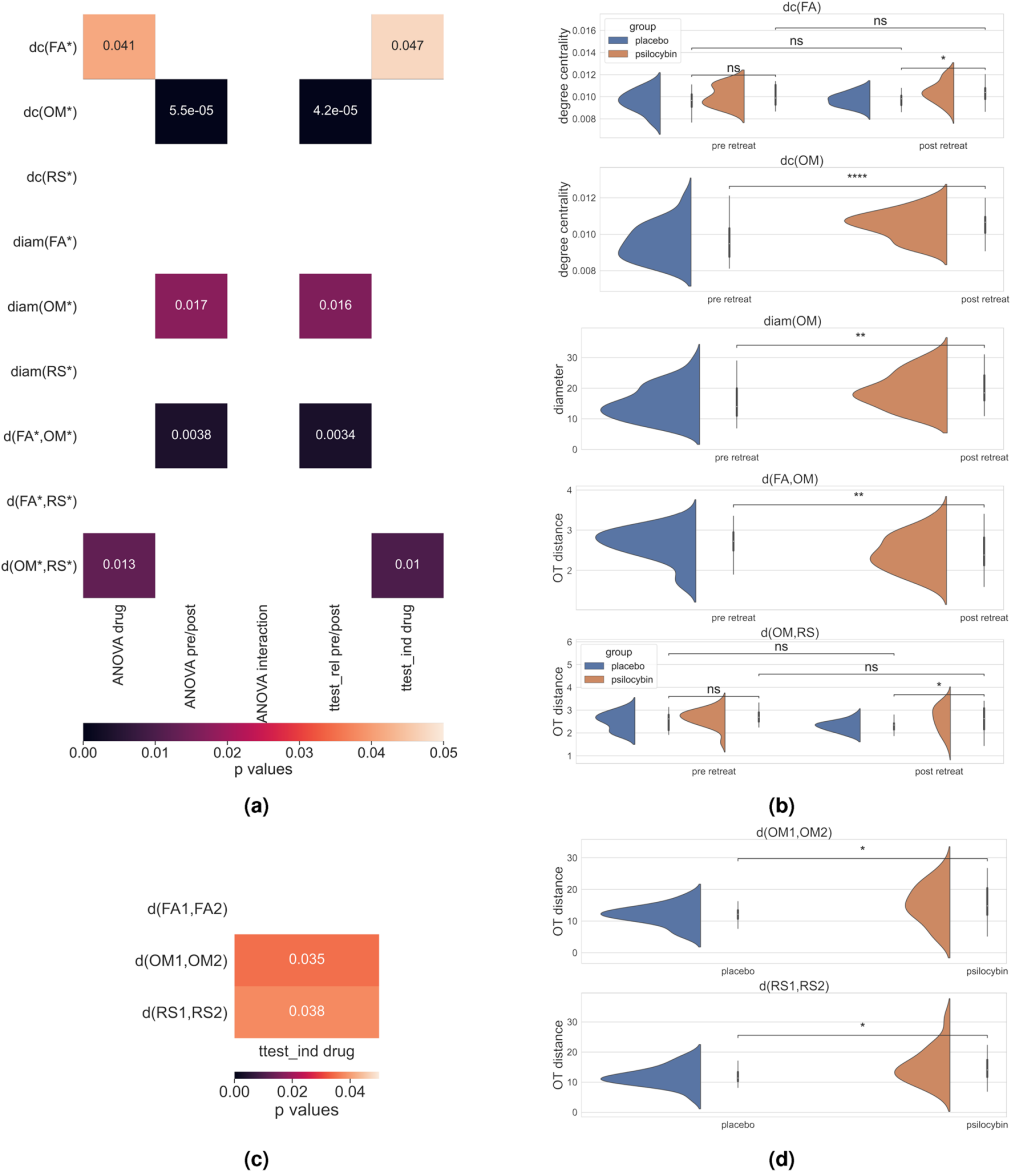
**(a)** A heatmap representing the statistical tests; colored boxes indicate significant differences according to the *p* values annotated and according to the color bar (multiple comparison correction of the first six rows by $$*3$$, see Methods). For notationsin the rows, see Table 1. The first three columns represent the ANOVA tests with drug (psilocybin vs. placebo), retreat (pre vs. post), and interaction effects of drug*retreat, respectively. The fourth column represents the paired t tests comparing pre- vs. postretreat deata over the whole dataset. The fifth column represents the colorred results of the independent t tests of psilocybin vs. placebo postretreat. **(b)** Distributions of the graph measures plotted according to the significance tests in 2a. Notations; ns: not significant or $$p> 0.05$$, $$*: 1.00e-02< p <= 5.00e-02$$, $$**: 1.00e-03< p <= 1.00e-02$$, and $$***: 1.00e-04< p <= 1.00e-03$$. **(c)** Independent t tests for the drug effect on the OT distances before vs. after the retreat of the same meditation styles. **(d)** Significant (according to the t test in (c)) differences (placebo vs. psilocybin) in the distributions of the distances between OM1 and OM2 and between RS1 and RS2. The notations are the same as before.

**Figure 3:**
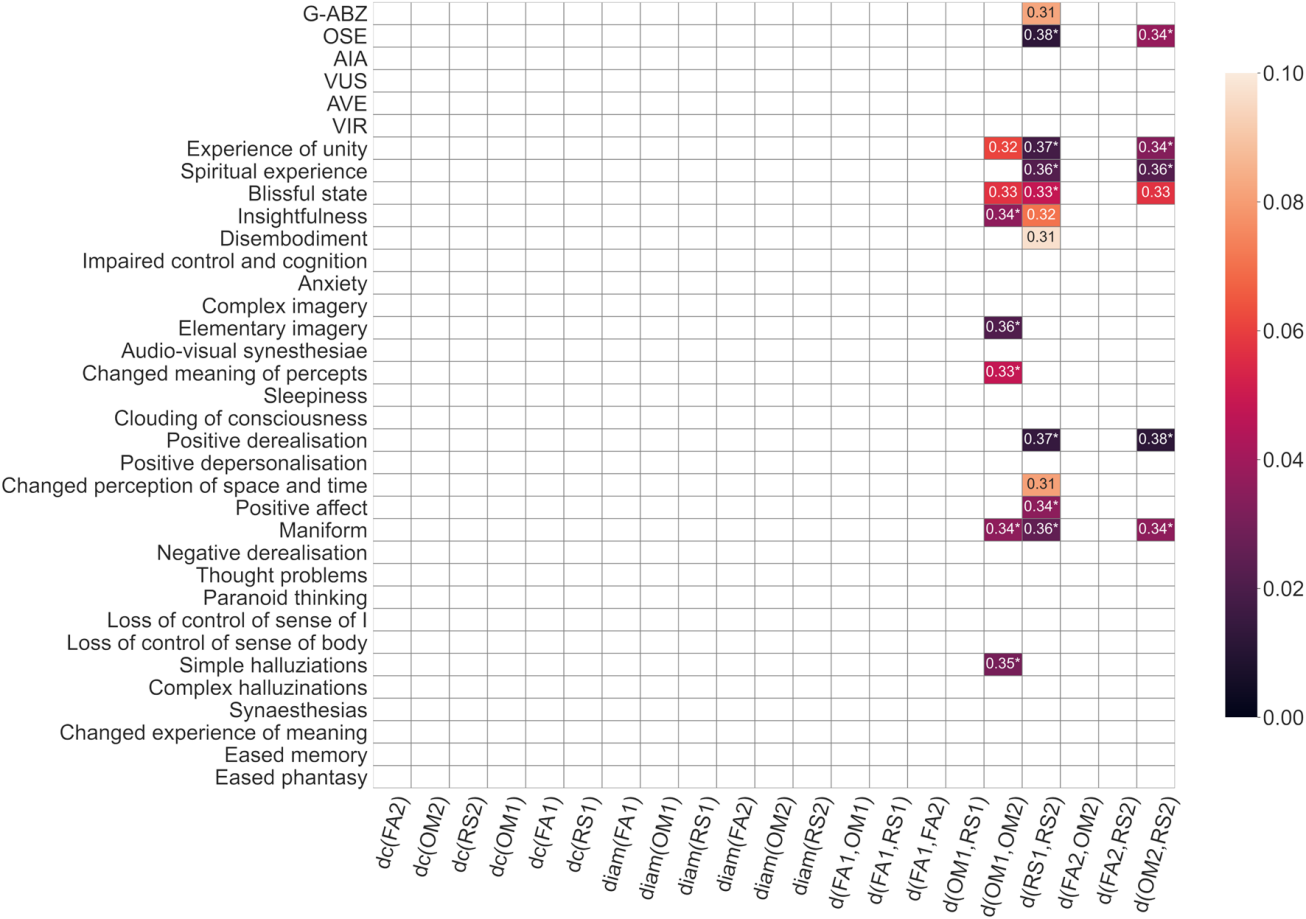
Kendal rank correlations and *p* values (according to the color bar, up to a *p* value of 0.1) of topological descriptors with rankings from the altered states of consciousness (ASC)^73^ questionaire, corrected for effective number of multiple comparison (*10, see Methods). Significant ($p<0.05$) correlations are marked by a $*$. The three distances (*d*(*OM*1, *OM*2), *d*(*RS*1, *RS*2) and *d*(*OM*2, *RS*2)) are strongly correlated (see Fig. 1c) and show significant drug effects (see Fig. 2).

The abbreviations used throughout this paper are shown in Table1. To analyze the topological landscape of meditative brain states at the subject level we computed topological descriptors for the Mapper shape graphs of all 36 subjects, including degree centrality, closeness centrality and diameter for each experimental condition FA, OM, and RS), OT distances for each pair of experimental conditions within the same day of measurement (e.g. d(OM1,FA1)), and OT distances between pre- and post-intervention under the same experimental conditions (e.g. d(OM1,OM2)). Subsequently we compared these topological descriptors for the drug (psilocybin vs. placebo) and retreat (pre vs. post) effects (Fig. 2) and identified correlations with psychometric measures (Fig. 3). For OT distances before vs. after treatment under the same experimental conditions (e.g. d(OM1,OM2)) we analyzed only the drug effect, as the retreat effect is intrinsic to this measure.

An example of a Mapper shape graph for one representative subject is provided in Fig. 1a. Although the appearance of Mapper shape graphs varies for each subject, they share topological principles of organization that are similar for both groups and hence show the effect of meditation. Additionally, alterations in specific topological measures (within a similar overall topology) can be observed and linked to psilocybin induced alterations of consciousness. Figure1b provides a schematic representation of the average topological organization of Mapper shape graphs across all 36 subjects. We observe a shift of all three mediation states (i.e. nontrivial *d*(*OM*1, *OM*2), *d*(*RS*1, *RS*2), and *d*(*FA*1, *FA*2), visualized by the shift of the triangle *FA*2-*OM*2-*RS*2 with regard to *FA*1-*OM*1-*RS*1) in both groups due to the retreat, which was greater than the differences between mediation styles (e.g. OM vs. FA) within the same day of measurement. (For example, *d*(*OM*2, *FA*2) is shorter than *d*(*FA*1, *FA*2).) Moreover, the shift in the open monitoring and resting state was significantly greater in the Ppsilocybin group. (Fig. 2c, which also provides all p values.) Most interestingly, the main significant differences between the psilocybin and placebo groups were changes in and variances in OT distances (between OM and RS) that were related to psilocybin-induced positive derealization and similar psychometric ratings. Since the other topological features were stable across the groups, this finding shows that the model in Fig. 1b reliably describes the effect of the retreat, specifying the psilocybin-induced alterations of the topology and linking them to psychometric experience ratings. Notably, the model is more sensitive to the overall effect of the retreat on resting-state fMRI (rsfMRI) changes (i.e., in all three states FA,OM, and RS) than to the differences between meditation styles (on the same day of measurement). Moreover, our results exemplify (with the help of extensive methodological development in^8,9^) the potential of these methods to provide novel topological brain markers related to meditation and psychometric experience of altered states of consciousness.

**Figure 4:**
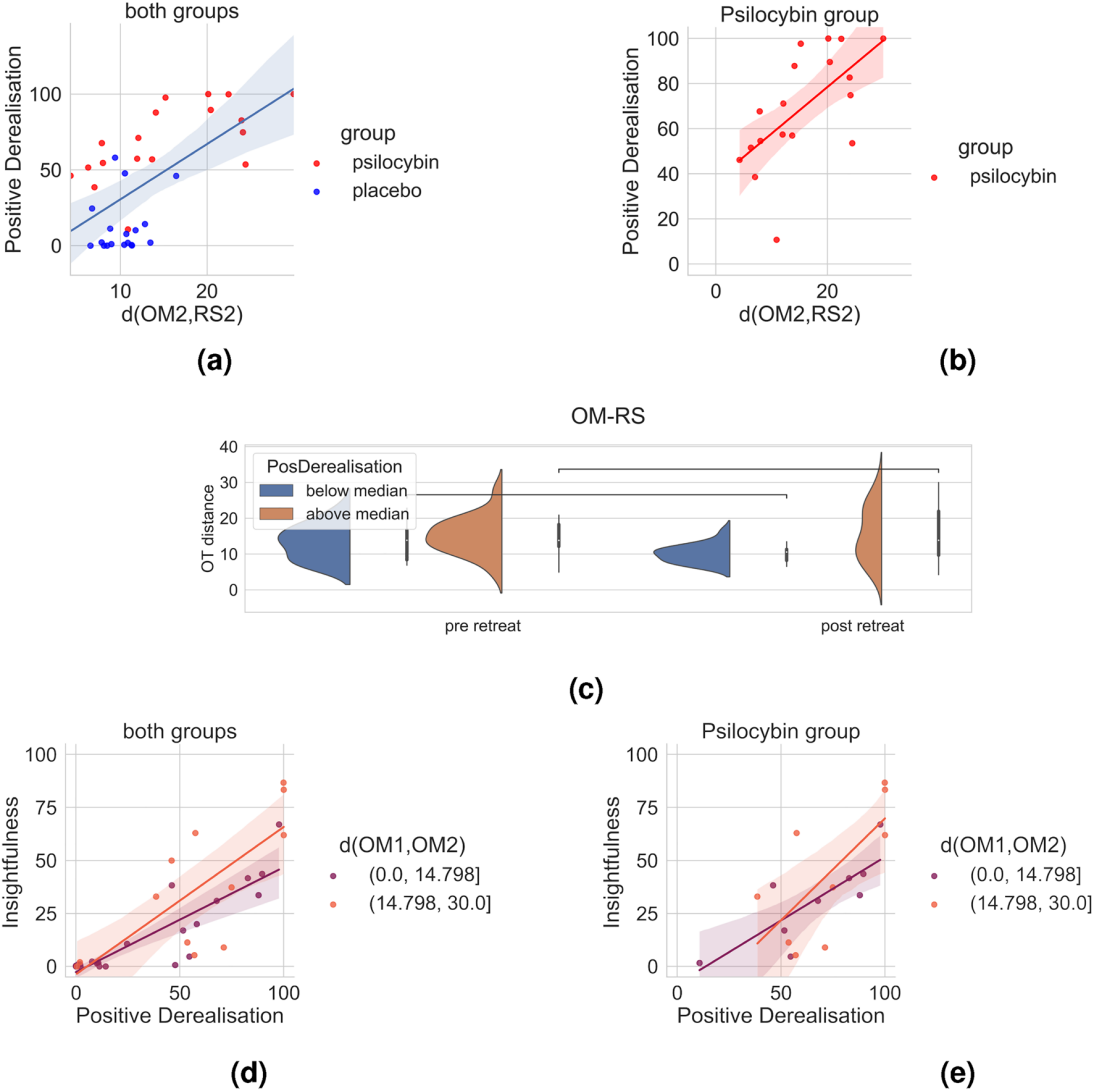
**(a)** Correlation between *d*(*OM*2, *RS*2) and positive derealization. The skewness in the data was noticeable, and the placebo group tended to accumulate toward one end of the plot. Notably, the results of the mixed linear model are stronger since they show a retreat effect and a combined effect of retreat and positive derealization. **(b)** Correlation between *d*(*OM*2, *RS*2) and positive derealization for the psilocybin group. **(c)** Significant decrease in $d(OM*,RS*)$ due to retreat for those subjects with positive derealization ratings below the median. **(d)** Visualisation of the linear model for the dependent variable insightfulness with two explanatory variables (for the whole group). Positive derealization and *d*(*OM*1, *OM*2) together provide better predictions of insightfulness than does positive derealization alone. The data were split into two equal groups at their median value (indicated by the two colors and the values are in brackets on the right). **(e)** Same as 4c but for the psilocybin group.

**Figure 5:**
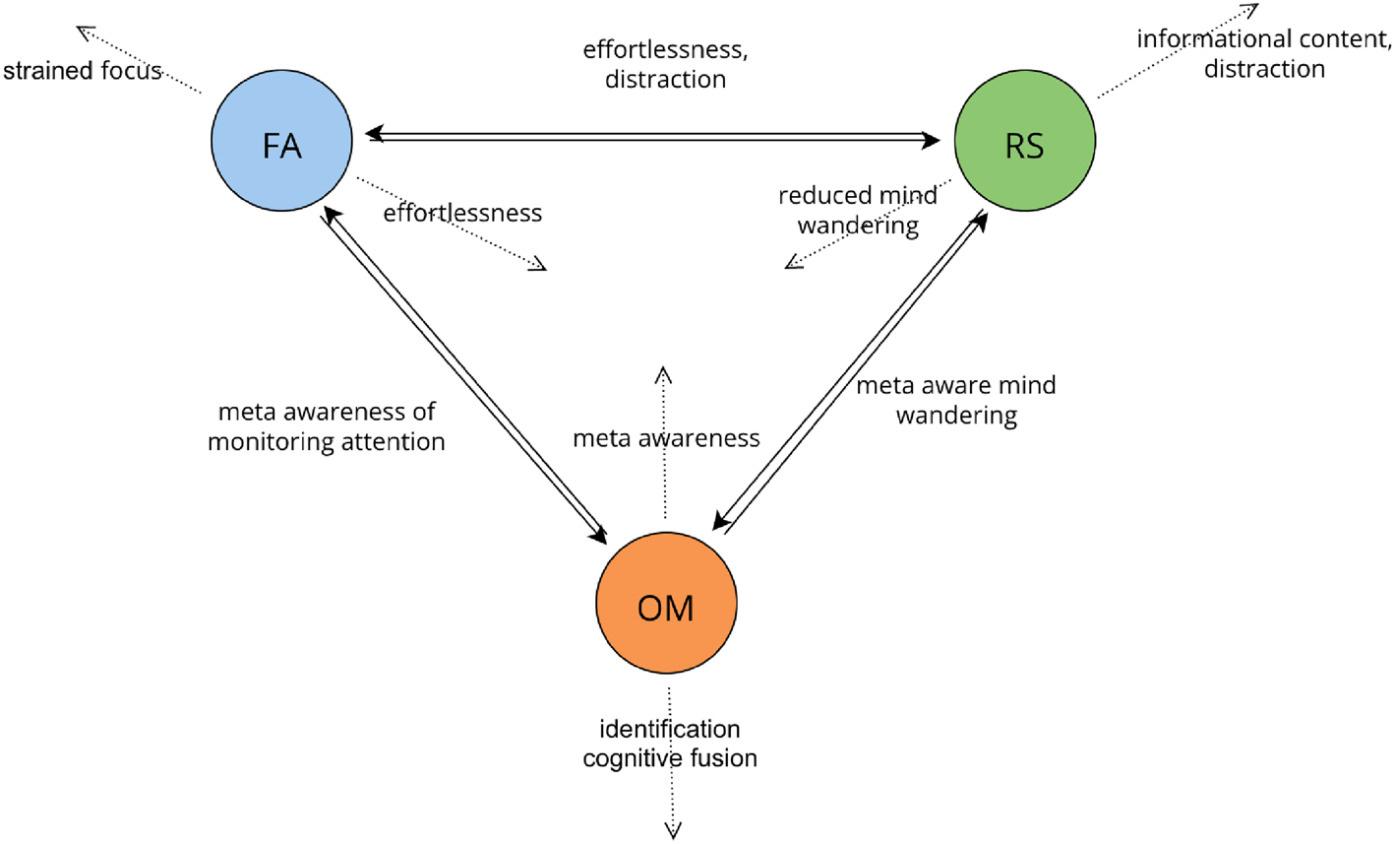
A hypothetical topological model of core phenomenological features and their relationships with mindfulness-related practices. Here, the distance between the nodes represents the topologically measured OT distance in the landscape of meditative states (i.e. Mapper shape graph of FA, OM and RS) and reveals relationships and interactions (overlap and similarity) of mindfulness-related practices at the level of brain activity. This perspective may provide insights into how changes in consciousness and perception during meditation or psilocybin-assisted mindfulness practices translate into alterations in the topological landscape and allow further exploration into the sometimes complementary and opposing yet potentially synergistic effects between mindfulness-related practices and the phenomenology of psychedelic experiences. Hypothetically, certain changes in perception, cognition and consciousness are associated with increased OT distances between FA, OM, or RS (i.e., less interaction, overlap, or similarity), which are represented by arrows pointing away from the center. Conversely, other changes in perception, cognition and consciousness may be associated with decreased OT distance between FA, OM, or RS (i.e., more interaction, overlap, or similarity), which are represented by arrows pointing toward the center. This theory is consistent with our findings (Figs. 2 and 3). Decreased $d(FA*,OM*)$ might be an indicator of increased meta-awareness while monitoring attention and distraction. Indeed, we observed that *d*(*FA*, *OM*) decreased due to the retreat. Similarly, a decreased $d(RS*,OM*)$ might be an indicator of meta-awareness of mind wandering or informational content, which is supported by the observation that $d(RS*,OM*)$ significantly decreased due to the retreat in participants with lower ratings of positive derealization (Fig. 4c). The correlation of $d(RS*,OM*)$ with positive derealization supports the idea that increased informational content increases the OT distance between RS and OM. While increased effortlessness of focus presumably decreases $d(FA*,RS*)$, decreased distraction increases $d(FA*,RS*)$). Notably, this could be a plausible explanation for our observation that $d(FA*,RS*)$ did not change pre- or postretreat since the two effects cancel each other out.

The main topological organizations of meditation are visualized in Fig. 1, statistics are summarized in Figs. 2 and 3. Main results are further emphasized in Fig. 4 and interpreted in Fig. 5. First, we found a significant difference in the OT distance *d*(*OM*2, *RS*2) according to drug treatment (placebo vs. psilocybin), with higher median values and variance in the psilocybin group. Correlation analyses with altered states of consciousness ratings showed that the OT distance *d*(*OM*2, *RS*2) was positively associated with positive states of consciousness, such as positive derealization (Fig. 4a and b), which are commonly enhanced by psilocyin (Fig. 3). Moreover, we observed a significant retreat and interaction effect of positive derealization and retreat on $d(OM*,RS*)$ for the whole group as well as for the psilocybin group. We found that $d(OM*,RS*)$ decreased significantly due to retreat in those subjects whose positive derealization was below the mean (Fig. 4c). However, psilocybin increased positive derealization, which was linked to higher *d*(*OM*2, *RS*2) compared to placebo (Fig. 2b). Notably, these observations on $d(OM*,RS*)$ were not due to acute effects of psilocybin, as the fMRI scans taken post-retreat (i.e. 48 hours after drug administration). Moreover, insightfulness was strongly correlated with psilocybin-induced positive derealization during meditation, and the latter relation tended to be stronger for higher *d*(*OM*1, *OM*2) (Fig. 4c and d) Second, we observed that the optimal transport distances *d*(*OM*1, *OM*2) and *d*(*RS*1, *RS*2) were significantly greater in the psilocybin group than in the placebo group (Fig. 2c). Third, we observed that FA and OM became more similar (i.e., decreased *d*(*FA*, *OM*)) after the retreat across all subjects (Fig. 2b). Finally, the degree centrality, closeness centrality and diameter of the OM increase over the retreat for both groups (Fig. 2b).

### Statistics

First, for each topological desriptor a two-way repeated-measures ANOVA was performed, with group as between-subject and retreat as within-subject factor. Post hoc tests were performed in cases of significant main effects. For $d(*1,*2)$ only paired t tests were performed to examine differences in drug effects. Because the psychometric data were skewed (Fig. 4a), Kendal rank correlations and p values of topological descriptors with measures from the altered states of consciousness (ASC) questionnaire^73^ were computed (Fig. 3) and corrected for 10 effective numbers of comparisons (see Methods). The graph measures were strongly correlated (Fig. 1c), as are many of the scales of the ASC questionnaires^73^.

We fitted a mixed linear model (for the whole group) to the outcome variable $d(OM*,RS*)$; with pre/postretreat and positive derealization as fixed effects and subject as a random effect. We then fitted a similar model to the psilocybin group. Additionally, we fitted linear models (for the whole group and for the psilocybin group) to the outcome variable insightfulness, with positive derealization and *d*(*OM*1, *OM*2) as explanatory variables. Finally, with a median split we verified that $d(OM*,RS*)$ decreased for low positive derealization.

#### Modulation of optimal transport distances after psilocybin-assisted meditation retreat

A significant group effect on $d(OM*,RS*)$ was found, as revealed by mixed ANOVA ($F_{1,34}= 6.9$, $p = 0.013$). Independent t test of group differences for *d*(*OM*2, *RS*2) revealed a significant difference $t_{20.6} = -2.7, p = 0.010$), with increased variance and mean *d*(*OM*2, *RS*2) in the psilocybin group compared to the placebo group. However, overall $d(OM*,RS*)$ tended to decrease due to the retreat (Fig. 2b). Moreover, a significant session effect on $d(OM*,FA*)$, as revealed by mixed ANOVA ($F_{1,34} = 9.7$, $p= 0.0038$), was found. $d(OM*,FA*)$ decreased significantly, as revealed by paired t test ($t_{35}= 3.14$, $p = 0.0034$). No significant effects were found for *d*(*FA*, *RS*).

#### Psilocybin enhances the retreat-induced changes in *OM* and *RS*

As revealed by independent an t test, the mean OT distance *d*(*OM*1, *OM*2) was significantly longer with greater variance (Fig. 2c) in the psilocybin group than in the placebo group ($t_{25.7} = -2.2, p = 0.037$). The results for *d*(*RS*1, *RS*2) were similar ($t_{26.6} = -2.2, p = 0.04$).

#### The optimal transport distance $d(OM*,RS*)$ was associated with psilocybin-induced positive derealization in meditation

Positive derealization showed the strongest positive Kendal correlation with *d*(*OM*2, *RS*2) ($r = 0.379$, with $p = 0.00118$ and corrected $p = 0.0118$). The first mixed linear model ($d(OM*,RS*)$ $\sim$ positive derealization*session) revealed a significant retreat effect (t = $-2.624$, p = 0.0129) and a significant interaction effect of retreat and positive derealization (t = 2.315, p = 0.0268). Its marginal and conditional R-squared values were 0.267 and 0.380 respectively. Similarly, the analogous mixed linear model for the psilocybin group revealed a significant retreat effect (t = $-2.293$, p = 0.0358) and a significant interaction effect between retreat and positive derealization (t = 2.446, p = 0.0264). Its marginal and conditional R-squared values were 0.2595 and 0.4869, respectively. A median split of positive derealization over the whole dataset and subsequent t test showed a significant decrease (t = 2.3348, p = 0.03207) of $d(OM*,RS*)$ due to the retreat for those subjects with low positive derealization and no significant effect on the other group.

#### Insightfulness through positive derealization in meditation is enhanced by greater dissimilarity of the OM before vs. after retreat

Insightfulness and positive derealization were strongly correlated (Pearson $r= 0.82$, $p=6.56e-10$). The linear models (for the whole group and for the psilocybin group) with the explanatory variables positive derealization and *d*(*OM*1, *OM*2) and the dependent variable insightfulness were both significant (whole group: $F_{3,31} = 25.1$, $p = 1.963e-08$ and psilocybin: $F_{3,13}= 7.061$, $p = 0.00464$) and stronger (higher adjusted R-squared) than the model with only positive derealization as the explanatory variable. For the whole group, the multiple and adjusted R-squared values were 0.7084 and 0.6802, respectively, and the interaction effects of positive derealization and *d*(*OM*1, *OM*2) showed a trend toward significance ($p =0.075$). Similarly, for the psilocybin multiple and adjusted R-squared are 0.6197 and 0.5319 respectively, and interaction effects of positive derealization and *d*(*OM*1, *OM*2) showed a trend toward significance ($p= 0.081$). The simple linear model of positive derealization vs. insightfulness marginally improved (regarding the goodness of fit, i.e., adjusted R value) by adding the variables “drug experience” or “number of meditation hours in lifetime”.

#### The degree centrality, closeness centrality and diameter of the OM increases after meditation retreat

The degree centrality of the OM showed a significant effect of retreat ($F_{1,34}=24.8$, $p_{corr} = 5.5e^{-5}$). A post hoc paired t test revealed a significant increase in the degree centrality of the OM ($t_{35}= -5.04$, $p_{corr} =4.2e^{-5}$). Similarly, the closeness centrality of the OM showed a significant effect of retreat ($F_{1,35} = 20.8$, $p_{corr} =.9e^{-4}$. A post hoc paired t test revealed a significant increase in the closeness centrality of the OM ($t_{35}= 4.6$, $p_{corr} = 1.5e^{-4}$). The diameter of OM also showed a significant effect of retreat ($F_{1,35} = 8.7$, $p_{corr} =0.017$). A post hoc paired t test revealed a significant increase in the diameter of the OM ($t_{35}= 2.97$, $p_{corr} = 0.016$).

#### Other effects

The degree centrality of FA after retreat was significantly greater in the psilocybin group than in the placebo group. However, the effect was close to nonsignificant, and group differences were already observed before the retreat (Fig. 2b).

## Discussion

This is the first brain imaging study combining topological data analysis (TDA) of BOLD signal fluctuations (fMRI) in a sample of thirty-six experienced meditators before and after a 5-day psilocybin-assisted meditation group retreat. This is also the first study of the modulatory effects of psilocybin on the topological landscape of meditative brain states using a novel, previously established approach^8^ based on Mapperand TDA. We found that organizational principles in the topological landscape of meditative states were associated with meditation retreat effects and that specific topological measures correlate with enhanced positive experiential effects of psilocybin combined with meditation. The latter processes reflect a modulation of the topological properties in the landscape of meditative brain states, which in turn can be used to better understand the beneficial psychological processes underlying synergistic effects of psilocybin and meditation.

The OT distance provides a tool for studying the relationships and interactions of different meditation states in terms of the similarity and overlap of their whole-brain activation patterns. For example, if two different meditation states correspond to similar combinations of activated brain regions, the OT distance between them is small. In contrast, if complementary regions are activated during the two meditation states, the OT distance increases. The OT distances can then potentially be related to changes in perception, cognition and consciousness. Conceptually, the OT distance can be thought as a measure of information flow on the level of brain activity between two distributions of mediation states, i.e. labels on the nodes in a Mapper shape graph. This can be illustrated by the analogy of optimizing the transport cost of a product from a collection of suppliers to a collection of vendors; the transport cost is optimized while taking into account the underlying distributions of suppliers and vendors as well as all pairwise distances between them. Hence, the optimal transport distance is a measure that not only is a distance but also entails a rich geometric structure in the space of the underlying probability distributions^74^.

Overall, we observed a shift in rsfMRI activity in all three states - open monitoring, focused attention, and resting state - due to retreat (i.e., nontrivial OT distances *d*(*OM*1, *OM*2), *d*(*RS*1, *RS*2), and *d*(*FA*1, *FA*2)). These effects were greater than the OT distances between different mediation states within the same day of measurement (e.g. *d*(*OM*2, *FA*2) is shorter than *d*(*FA*1, *FA*2)). This effect is illustrated by the shift of the triangle FA2-OM2-RS2 with respect to FA1-OM1-RS1. This is most likely due to an overall (i.e., affecting all states, [FA, OM and RS]) change in rsfMRI activity due to the mediation retreat. Hence, the method is more sensitive to the overall retreat effect on rsfMRI activity than it is to differences between meditation states. The characteristics of the topological landscape were relatively stable across the groups, revealing the reliable effect of the retreat. Most interestingly, specific topological alterations (discussed below) in the psilocybin group, can be linked to positive psychometric ratings (see Fig. 3 and the next paragraph). Together, these findings indicate differential effects of psilocybin on the topological architecture of brain networks underlying different meditation states. Moreover this approach could be used to extract topological markers (using graph theoretic measures) for identifying beneficial psychological processes underlying the synergistic effects of psilocybin and meditation.

Among the 35 ASC ratings that showed significant correlations with one of the distances, insightfulness is the most likely to have therapeutic potential because it can serve as a reference point for reflecting and integrating positive experiences. For topological interpretation $d(OM*,RS*)$ is most interesting because it describes the relation between two different states of consciousness and how the latter is affected by meditation and psilocybin. It was hypothesized^75^ that oceanic self-boundlessness (OSB) , of which positive derealization is a subscale, would predict outcomes of psilocybin-assisted therapy for treatment-resistant depression. Combined effects of mediation and psilocybin-induced alterations in consciousness, such as positive alterations in the perception of the external world and positive derealization, were associated with greater OT distances between OM and RS postretreat (Fig. 3). Moreover, positive derealization had the most significant correlation with *d*(*OM*2, *RS*2) and was most prominent in the correlation matrix. Hence, in our analysis, we focused on investigating the effect of the retreat and the drug on positive derealization and, subsequently, how positive derealization may be related to insightfulness. First, we found that psilocybin supports insightfulness in meditation and that insightfulness correlates with greater changes in open monitoring meditation (i.e., *d*(*OM*1, *OM*2)). Moreover, insightfulness correlated with positive derealization (which was linked to increased OT distance between OM and RS, i.e., $d(OM*,RS*)$) , and notably , the latter correlation was stronger for greater changes in OM (i.e., greater *d*(*OM*1, *OM*2)). Conversely, we observed that OT distances between OM and RS (i.e., $d(OM*,RS*)$) decreased in the subgroup of participants with low ratings of positive derealization (Fig. 4c) due to the retreat. Together , these findings could indicate that enhanced aperture and meta-awareness (which are commonly trained during OM) combined with psilocybin-induced positive derealization (through opposite effects on $d(OM*,RS*)$) fosters insightfulness.

Second, we showed that meditation increased the degree-centrality, closeness-centrality, and diameter of the OM, which could be another indicator of increased aperture and meta-awareness (for a a conceptual description and discussion Figure below). Third, a five-day meditation retreat for experienced mediators decreased the OT distance between OM and FA, which could indicate increased meta-awareness of monitoring attention.

As apparent from previous analyses of the same dataset, psilocybin administered in a retreat setting supported by mindfulness practices, such as OM, significantly potentiated felt states of ego dissolution compared to the placebo; in particular, oceanic self-boundlessness (OSB) increased, of which insightfulness and positive derealization were substantial lower-order subscales^7^. Furthermore, persistent positive effects after the meditation retreat were positively correlated with OSB scores during psilocybin administration^7^.

A hypothetical topological model (Fig. 5) of detailed experiential features in mindfulness-related practices delineates the interpretation of our results. Figure 5 aims to explain how changes in perception, cognition, and consciousness may affect OT distances; in a way consistent with our findings. We start by identifying experiential similarities and differences between MSs and how they might affect OT distances. Enhanced OM (e.g., through practice) involves an increase in various phenomenological dimensions, such as increased meta-awareness, clarity, aperture, and dereification^76^. Enhanced FA (e.g. through practice) involves phenomenological dimensions such as increased focus, decreased distraction, increased effortlessness, and increased stability and clarity^76^. In direct contrast, a resting state typically involves mind-wandering, which is associated with low meta-awareness, clarity, aperture, and dereification^76^. Notably, what distinguishes RS from OM is meta-awareness of the ongoing experience, and mind-wandering^19^. However, as suggested by previous studies^5,77,78^ it is expected that resting state brain activity is also substantially affected by 5-day meditation practice. In fact, as meta-awareness is the main target of mindfulness-based practices, which were trained during the retreat, an increase in meta-awareness capabilities over the retreat in all conditions (FA, OM, and RS) is plausible. Moreover, the increase in meta-awareness observed under all conditions is consistent with our findings, as explained below and in Fig. 5. Notably, certain changes in consciousness and perception may have effects on different MSs that cancel the effect on the level of their OT distances (Fig. 5, e.g., the effect of meditation and positive derealization on $d(OM*,RS*)$).

The profound modulatory effects on consciousness, perception and cognition^7^ observed after psilocybin administration in our dataset are known to increase the entropy of spontaneous brain activity (examples include^51–55^; for an overview see^6,56^), which is hypothesized to indicate the informational richness of conscious states^6^. Although the precise effect of meditation on the entropy of spontaneous brain activity is not well understood, it is plausible that mediation combined with psychedelics, such as psilocybin, is characterized by brain states with greater entropy than meditation combined with a placebo. Together with our results, these findings suggest that a psilocybin-induced state of enriched informational content manifests as a greater OT distance between OM and RS (compared to that in the control group), which persists even beyond the retreat, potentially contributing to the increased levels of insightfulness when combined with open monitoring meditation training (i.e. increased meta-awareness). This might indicate that increased entropy due to psilocybin benefits from a counterbalancing effect of meditation to prevent the brain from going beyond a state of criticality where the ability to consciously reflect is impaired. According to the entropic brain hypothesis^6^, the combined effects of psilocybin and meditation could be described as divergent yet synergistic.

Conceptually, the increased degree and closeness centrality of the OM postretreat (represented by the central position of the OM2 in Fig. 1b) indicate that OM postretreat receives/exchanges information (with other MS in the Mapper shape) more frequently and at higher speeds (^9,11^). As meta-awareness is the ability to note the content of one’s current mental state^5^, increased closeness centrality of the OM observed across subjects could represent a neural correlate of increased meta-awareness. Aperture might be interpreted as the ability to bring into awareness more distinct contents of one’s current mental state^5^. Hence, the increased degree centrality of the OM observed across subjects may bea neuronal surrogate marker for increased aperture. Furthermore , as the diameter of the OM measures the maximum dissimilarity of brain activity during OM, an increased diameter of the OM postretreat (represented by the diameter of the OM2 node in Fig. 1b) might indicate broader diversity of the content of one’s mental state during OM. In summary, these findings support the idea that meditation practice enhances OM by increasing meta-awareness and the aperture of the ongoing stream of consciousness, including a greater variety of mental contents and mental states^19,48^.

We conclude that, compared with meditation alone, psilocybin alters the perception of the external world, presumably by increasing informational richness, which is reflected by increased OT distances between OM and RS postretreat . Moreover, the retreat effects on open monitoring meditation (i.e., OT distance between OM before vs. after), which presumably indicates meta-awareness and aperture enhanced through meditation practice, reinforce insightfulness gained through psilocybin-induced positive derealization. In other words, meta-awareness may serve as a common mediating factor, that potentially explains how the altered state of consciousness induced by psilocybin enhances insightfulness in experienced meditators.

The method has been extensively tested for reliability^8,9^; however, different dimension reduction techniques have been used. Notably, the approach is novel and under rapid development^8–11,79^. The specific dimension reduction method UMAP^80^ may have an effect on the results. The fMRI scanning durations in our dataset were comparatively short, potentially limiting definitive conclusions^9^. However, the total length of the scans was comparable to that of other studies with the same method that correlated task performance with OT distances^11^. We obseved that the topology (Fig. 1b) was relatively stable across the groups with differences that correlated with psilocybin-induced psychometric alterations. This finding supports the reliability of our results, despite the short scanning duration, the concatenation of the data and the many confounding factors in the fMRI data.

Furthermore, while findings from previous studies^11^ on OT distance are based on dataset s with different levels of task performance, it is more challenging to define and control task performance in meditation, especially during rsfMRI scanning sessions (e.g., loss of concentration or distraction). However, since there is evidence^81^ that novice practitioners can reliably report their experience, the reliability of experienced practitioners on meditation features can be assumed if they are trained to acknowledge and perform them. Hence a limitation in our dataset is the lack of a control group of naive or novice meditators. It would also be interesting to include a group of participants who take the psilocybin without participating in the meditation retreat. Moreover, 7 minutes could be too short to reach a significant meditation state effect, but the instructions during scanning were intended to simulate a natural way of entering into focus attention and open monitoring meditation^7,72^.

Meditation (such as OM) and psychedelics are both associated with reduced activation in the DMN and increased functional connectivity between DMN structures and certain task-positive networks^43,46,48–50^. As an exception, there is preliminary evidence of increased anti-correlation between the DMN and TPN during FA^43,82^. Various other brain networks, such as alerting, salience and orienting networks, were associated with meditation practice^83,84^. Hence, annotating intrinsic brain network activity on the Mapper shape graphs (similar to resting states^9^) could lead to neurophysiological explanations of the organizational principles observed in the Mapper shape graph of meditative states. Furhtermore, annotating nodes in the Mapper shape graph with entropic measures in regions of interest in the brain may reveal novel insight into how entropy changes the flow of information in the brain. For example, this could lead to a better understanding of how enriched informational content could affect the OT distance between OM and RS.

To better understand the phenomenological or psychological implications of the organizational principles and potential modulations, this approach could be combined with more comprehensive phenomenological data. While most current experiential measurements are based on psychometric questionnaires (such as so-called “thin” measures), more recent studies have increasingly made a case for using “thicker” methods of phenomenological investigations that provide more refined and authentic qualitative accounts of experience^85^. After this so-called neurophenomenological approach has originated as an offshoot of enactive cognitive science^86,87^, it exhibited increasing influence on research on nonordinary states of consciousness, including meditation, psychedelics, and hypnosis^88^. This neurophenomenological approach combining the topological analysis of third-person neuroimaging data and an in-depth phenomenological analysis of rigorous first-person experience reports might represent a promising direction for future research.

## Methods

### Dataset

The study was registered ClinicalTrials.gov with registration number NCT03736980, and the date of registration was 9/11/2018. The study was approved and published according to the CONSORT 2010 guidelines^7^. The study was approved by the Cantonal Ethics Committee of the Canton of Zurich (KEK-number 2013-0473) and the Swiss Federal Office of Public Health. All participants provided written informed consent according to the Declaration of Helsinki. The use of psilocybin according to the study protocol was authorized by the Swiss Federal Office for Public Health, Department of Pharmacology and Narcotics, Bern.

Data from 38 healthy, experienced meditators (23 males, mean age 51.66 ± 8.32) were analyzed^7,72^. Written informed consent was obtained prior to study enrollment. The participants were matched for age, sex, previous meditation experience, and dispositional mindfulness and randomly assigned to the psilocybin or placebo group. The active and control groups included 18 and 18 subjects, respectively. Two subjects were excluded from the analysis (one due to a detected exclusion criterion for fMRI and one due to technical problems with reconstruction of the Mapper shape graphs).

Participants underwent a 5-day silent meditation retreat, known as *sesshin* in the Zen tradition (for details regarding the study procedures and the setting, Fig. ^7,72^). On the fourth day at 10:30 a.m., psilocybin (315 $\mu \hbox {per}\,\hbox {kg}$ body weight; absolute dose, 21.82 ± 3.7 mg) and a placebo (lactose) were administered in a randomized, double-blind, placebo-controlled design during the daily meditation routine . fMRI scans were collected the day before and the day after the retreat. The 21-minute scans consisted of three 7-minute blocks, each with eyes closed, in a fixed order, approximating a naturalistic meditation practice: resting state (RS), focus attention meditation (FA) and open awareness or open monitoring meditation (OM). Participants were experienced meditators from different traditions with no or little previous experience with psychedelics. During the resting state, subjects were instructed to rest but not meditate. The instructions for focused attention were to focus on the breath and return once distraction was noticed. Finally, the instructions for open awareness meditation were to observe whatever arose in the perception without clinging or attachment. Focus attention meditation was introduced as a way of entering open monitoring meditation. The order of meditation instructions provided during scanning was intended to simulate a natural way of entering into open monitoring meditation. For more details see^7,72^. The order in which the participants were scanned was random; however, to prevent time-of-day effects, the time of the pre- and postintervention measurements were the same plus or minus 3 hours.

### MRI data acquisition and scanning parameters

Anatomic and functional fMRI data were recorded using a 3T Philips Achieva MRI scanner with a 32-channel SENSE head coil. High-resolution T1-weighted gradient-echo images were acquired for structural reference at the beginning with a 3D field echo sequence (TR = $9.3\,\hbox {ms}$; TE = $4.6\,\hbox {ms}$; FOV = $240\,\hbox {mm}$; flip angle = $8^{\circ }$; in-plane resolution = $1\times 1 \times 1 \, \hbox {mm}$; 160 sagittal slices). The functional data were recorded with a $T2^{*}$-weighted echo-planar functional scan (slice-thickness = 4$\,\hbox {mm}$, no gap; 32 slices; TR = $2000\,\hbox {ms}$; TE = 35$\,\hbox {ms}$; FA = $82^{\circ }$). The raw fMRI data were acquired with a voxel size of 2.75 $\times 2.75\,\hbox {mm}$ and a slice gap of $4\, \hbox {mm}$. After normalization, the images had a voxel size of 2 $\times$ 2 $\times 2\, \hbox {mm}$. Participants’ hearing was protected from the scanner noise with earplugs and headphones. Foam pads were used to reduce head motion (see also^7^).

### Preprocessing

We performed a standard preprocessing procedure with additional nuisance regression analysis, which is crucial for the Mapper algorithm^8^. We performed fMRI preprocessing steps using the Configurable Pipeline for the Analysis of Connectomes (C-PAC version 1.6.0, https://fcp-indi.github.io/). Initial preprocessing of the functional images involved slice time correction and motion correction using FSL mcflirt with a reference mean. Anatomical images were skull stripped using FSL BET and their tissues were segmented with FMRIB’s Automated Segmentation Tool (FSL FAST tool). Normalization was performed according to the usual three steps: first, the anatomical images were transformed to the common template space using the Advanced Normalization Tool (ANTS); second, the functional images of each participant were registered to their anatomic image using BB Registration and AFNI 3dAutoMask; and third, the functional derivatives were transformed to the common template space using T1 template registration. We chose the common 152 brain template maintained by the Montreal Neurological Institute (MNI,^89^). For the functional images, we used a resolution of 3 mm, and for the anatomic images we used a resolution of 2mm. Nuisance signal correction was performed on the data by regressing out 24 motion parameters obtained by motion correction using FSL MCFLIRT (the six motion parameters of the current volume and the preceding volume plus all the values squared). The mean time series from the white matter (WM) and cerebrospinal fluid (CSF) were corrected. The detrended PC method with five components of the aCompCor algorithm was used to extract linear and quadratic trends from WM and CSF. After normalization the images had a voxel size 2 $\times$ 2$\times$ 2 mm, and a temporal bandpass filter ($0.009 \hbox {Hz}<\hbox {f}<0.08 \hbox {Hz}$) was used, then spatial smoothing with Gaussian kernel (FWHM of 4mm) and z-scoring was performed .

### Background on TDA methods

The TDA-based Mapper has some similarity withtraditional non-linear dimension reduction methods but it uses topological concepts to maintain information on the shape of the data in native space, while sharing theother aforementioned common denominators of TDA methods.

Several Mapper software programs^90^ are available and an open source platform^10^ was recently designed with a toolkit to explore and visualize Mapper-generated graphs and their topological properties with built-in support for neuroscience^10^. A quantitative tool from computational optimal transport theory^74^, which uses the 1-Wasserstein distances^91^ was suggested to quantify pairwise similarity between annotations in Mapper shape graphs^11^.

### Mapper pipeline

We largely followed the approach of Saggar and colleagues^8,10^. After preprocessing (C-PAC), we used Nilearn to load the 4D NIfTI time series images and extract 2D ($\#$TRs $\times$ $\#$voxels) NumPy matrix representations for input to Mapper. This can be considered a point cloud of size $\#$ TRs in a *representational space* of dimension $\#$ voxels. The implementation of Mapper was performed using python package Kmapper^90^. This algorithm uses machine learning (ML) algorithms (dimension reduction and clustering); however it is distinct from conventional ML approaches, as dimension reduction used only as a *filter lens* to slice the data to perform clustering in the high-dimensional space. The following is an overview of the steps in the algorithm:

1. *Filtering the data into a lower dimension* We used *uniform manifold approximation and projection*^80^ (UMAP) for dimension reduction (*a filter function*), which then (after step 2) served as a *filter lens* to slice the data.
2. *Binning the data along the filter dimensions* The projected data (i.e. in the image of the *filter function*) were covered by overlapping bins. For each bin, we considered the preimage (i.e., the set of all the data points mapped to that bin by the filter lens).The preimage is called the *slice* for the specific bin.
3. *Partial clustering* We used the density-based spatial clustering algorithmDBSCAN^92^. Clustering was performed independently for each *slice* in the high-dimensional space (as opposed to conventional ML methods). In this way, the high-dimensional data was compressed by grouping similar data points. Because the bins overlap, any two clusters in the slices of two adjacent bins can share data points.
4. **Graph construction** Each cluster in the high-dimensional space is represented by a node and nodes are connected by edges if the corresponding clusters share data points. This leads to a combinatorial representation of the topology of the data in a so-called *Mapper shape graph*.

After the graphs were computed, the visualization tools DyNeuSR^10^ combined with Nilearn and Nibabel were used to interactively explore the graphs. The nodes in the graph were labeled by pie charts corresponding to the meta data, i.e. the type of meditation or resting state.

### Mapper shape graphs, labeling, and subgraphs

After careful preprocessing of the data, for each of the 36 subjects, (*s*) and for each of the states (*l* $\in$ *MS*), we sliced the middle 100 frames (i.e. slice number 50 to 150) of the fMRI time series. In this way, we excluded most noisy slices. Moreover, we excluded transition times to obtain more reliable representations of the respective meditative or resting state of interest. For each subject, we then proceeded to concatenate the middle 100 frames of all six states *L*, resulting in a concatenated time series of 600 fMRI frames, corresponding to 21 minutes. The resulting concatenated time series forms a point cloud of size 600 in the *representational space* of dimension $\#$ voxels (see Section above). Using this point cloud as input, we prodeeded with the algorithm described above to generate one graph , $G_{s}$, for each subject. In this graph, each node represents a group of fMRI frames with similar activation patterns, and each frame is from one of the six labels corresponding to meditation states $MS = \{$FA1, OM1, RS1, FA2, OM2,or RS2$\}$. Using the interactive visualization tool reported in^10^, the resulting graph can be depicted in an html file, where each node has a mixed label from *L*, represented by pie charts.

### Details about the parameters

After performing dimension reduction with UMAP^80^, approximately $q = 60\%$ of the rectangular area of the image contained datapoints, and approximately $1-q = 40\%$ of the bins were empty. An easy computation shows that for *n* data points, *b* divisions in either dimensions and *p* overlap, the average number of data points per nonempty bin is $\frac{1}{1-q}\frac{n}{(b+p-bp)^{2}}$. An average of approximately 4 datapoints per nonempty bin worked well. This approach leaves enough flexibility for the algorithm to create enough edges between the nodes while still being able to detect the fine structures and details in the data, i.e., not building one graph of one big chunk of maximally, connected nodes. We choose 30 bins with an overlap of $50\%$ so that the estimated number of data points per node is 4.16. We used DBSCAN. The epsilon parameter was chosen to be the smallest possiblevalue , such that every data point has at least three neighbors^10^. We used the standard Euclidean metric.

### Graph measures

Table2 summarizes the graph measures and their abbreviations used for this study.

**Table 2:** Notations used for the graphs and graph measures.

Object	Description
$G = (N,E)$	A graph with nodes *N* and edges $E\subset N\times N$.
$d_{v}$	Degree (number of edges) of a node $v\in N$
$G_{s}$	Mapper shape graph for subject s
node labels; MS $= \{$FA1, OM1, RS1, FA2, OM2, RS2$\}$	Mediation states = conditions $\times$ sessions (pre and postretreat) $= \{$ FA, OM, RS $\}$ $\times$ $\{1,2\}$
$v\in N$ an *l* node	if *v* contains state *l* fMRI volumes
$\mu _l = \mu _l(G_s)$	Probability distribution of state *l* nodes in $G_s$, where $l \in L$.
$d_{OT}(\mu _l,\mu _k)$	Optimal transport distance between state *l* and *k* node distributions in $G_{s}$.
$cc(\mu _l)$	Weighted arithmetic mean of closeness centrality of *l* nodes in $G_s$.
$dc(\mu _l)$	Weighted arithmetic mean of degree centrality of *l* nodes in $G_s$.
*diam*(*l*)	Diameter of the set of *l* nodes in $G_{s}$.

We used the helper functions *closeness centrality*, *degree centrality*, *shortest path length* and the *Floyd-Warshall numpy* algorithm^93^, from the python package NetworkX to compute graph measures.

#### Optimal transport distance

The main tool used in OTs is the 1-Wasserstein distance, also known as the Earth Mover’s distance^91^. Given a graph *G* and its matrix of graph geodesic distances D and probability distributions p and q defined on the vertices of G, the OT distance quantifies the optimal cost of transforming one probability distribution (of node annotations in the graph) into another, thereby leading to a measure that is not only a distance but also entails a rich geometric structure on the space of probability distributions^74^. For the shape graphs (Figure also^11^), to obtain the probability distribution for a label $l \in MS$, we first take the proportion of state *l* data points for each vertex *v* of *G*, then normalize them to obtain a vector of length |*N*| with nonnegative numbers that sum to 1. With this procedure for two separate annotations by labels $l,k \in L$ yields two probability distributions *p* and *q*. Let $\Pi (p,q)$ denote the set of all joint probability distributions with marginals *p*, *q*. Each $\mu \in \Pi (p,q)$ is an $N\times N$ matrix with nonnegative entries summing to 1 and rows and columns summing to *p* and *q*, respectively. The 1-Wasserstein distance is defined as:

$$d_{{OT}} (p,q): = min_{{\mu \in \Pi (p,q)}} \sum\limits _{{i,j = 1}}^{N} D_{{ij}} \mu _{{ij}} .$$

#### Centrality and diameter

The closeness centrality of a node *v* is the reciprocal of the average shortest path distance from *v* to any reachable node in the graph. We defined the closeness centrality $cc(\mu _l)$ of state *l* as the weighted (by the probability distribution $\mu _l$) arithmetic mean of the centrality of *l* nodes. The degree centrality of a node *v* in a graph is the degree (= number of edges) of that node normalized by the maximum possible degree, which is $N-1$ if there are *N* nodes in total. We defined the degree centrality $dc(\mu _l)$ of state *l* as the weighted (by the probability distribution $\mu _l$) arithmetic mean of the degree centrality of *l* nodes. The diameter *diam*(*l*) is the maximum eccentricity of a state *l*, i.e. the longest of all shortest path lengths between any two *l* nodes.

### Altered states of consciousness rating scale

The Five Dimensional Altered States of Consciousness (5D-ASC) questionnaire^73^ was administered 360 minutes after the psilocybin or placebo intake as a retrospective measure of subjective effects. The 5D-ASC instrument is structured into 5 dimensions, and 11 empirically derived lower order scales (11-ASC)^94^. The first dimension is *oceanic self-boundlessness *(OSB),which is composed of the items “positive derealization”, “depersonalization”, and “altered perception of time and space”. The second dimension is *ancious ego dissolution *(AIA),which measures ego disintegration and the phenomenon of loss of self-control that evokes anxiety. The third dimension is *visionary restructuralization *(VUS), and its items correspond to the lower-order scales (11-ASC) ”complex imagery”, ”elementary imagery”, ”audio-visual synesthesia”, and ”changed meaning of percepts”. The fourth and fifth dimensions are *auditory alterations *(AVE) and *reduction of vigilance *(VIR) (n.b. translation from the German original may explain the slightly peculiar choice of terms). The 11 lower-order scales (11-ASC) are OSB:”experience of unity”, ”spiritual experience”, ”blissful state”, “insightfulness”, and “disembodiment”; AIA: ”impaired control of cognition”,and ”anxiety”; and VUS: ”complex imagery”, ”elementary imagery”, ”audio-visual synesthesia”,and ”changed meaning of percepts”.

### Statistics

#### Multiple comparison correction

The false discovery rate (FDR) for post-hoc t tests wascorrected using the Benjamini-Yekutieli procedure^95^ . For each condition (FA, OM, and RS) separately, we performed multiple comparison correction by $*3$ for the three measures (closeness centrality, degree centrality, diameter) on the nodes of the graph. We estimated the effective number of tests for comparison with the 35 psychometric measures (trip intensity, 5D-ASC and suborder scales) using the approach described in^96^, which is based on an approximation using the eigenvalues of the correlation matrix. The latter estimated the effective number of tests to be 10.

#### Effect of group by time on graph measures

For each of the measures (diameter, closeness and degree centrality, optimal transport distance) separately and each state (FA, OM, and RS) or tuple of states, we performd a two-way, mixed ANOVA with group as between-subject and session as within-subject factors, followed (if significant) by pairwise t tests, paired with respect to sessions and independent with respect to groups.

#### Correlation of phenomenology with graph measures

Pearson correlations of all the graph measures for all the conditions with the 35 psychometric measures (trip intensity and ASC) were computed and corrected for multiple comparisons. For the psychometric and graph measures with significant ($p<0.1$) Pearson correlations, we fitted mixed linear models, with session and the psychometric measure as fixed effects, subject as a random effect, and the outcome variable being the graph measure.

## Data Availability

The data and the code supporting the findings of this study are available from the corresponding authors upon reasonable request.
