# Author Correction: Psilocybin enhances insightfulness in meditation: a perspective on the global topology of brain imaging during meditation

**DOI:** 10.1038/s41598-024-59897-5

**Published:** 2024-04-23

**Authors:** Berit Singer, Daniel Meling, Matthias Hirsch-Hoffmann, Lars Michels, Michael Kometer, Lukasz Smigielski, Dario Dornbierer, Erich Seifritz, Franz X. Vollenweider, Milan Scheidegger

**Affiliations:** 1 Department of Adult Psychiatry and Psychotherapy, Psychiatric University Clinic Zurich and University of Zurich, Zurich, Switzerland; 2 Department of Psychosomatic Medicine and Psychotherapy, Medical Center - University of Freiburg, Freiburg, Germany; 3 Department of Neuroradiology, University Hospital Zurich, Neuroscience Center Zurich (ZNZ), University of Zurich and ETH Zurich, Zurich, Switzerland

Correction to: *Scientific* *Reports* 10.1038/s41598-024-55726-x, published online 26 March 2024

The original version of this Article contained an error in the name of author Berit Singer which was incorrectly given as Singer Berit.

The original Article has been corrected.

